# How does digital social media facilitate sports rehabilitation? Evidence from psychological recovery pathways and platform stickiness

**DOI:** 10.3389/fpsyg.2026.1700690

**Published:** 2026-02-20

**Authors:** Zhaoyu Liu, Qingyun Quan, Soohyun Kim

**Affiliations:** 1 Department of Sport and Healthcare, Namseoul University, Cheonan, Republic of Korea; 2 Sports Medicine Laboratory, Korea National Sport University, Seoul, Republic of Korea

**Keywords:** digital self-disclosure tendency, digital social media, platform stickiness, psychological recovery state, sports injury rehabilitation patients

## Abstract

**Objective:**

This study aims to explore how DSM influences the PRS of sports injury rehabilitation patients through a chain mediation mechanism, and how it further affects PS. In addition, it examines the moderating role of individuals' DSDT within this pathway.

**Methods:**

Questionnaire data were collected from 596 sports injury rehabilitation patients in China using a random sampling method. The measurement tools covered 10 psychological variables. A structural equation model was constructed using *AMOS 26.0* to perform chain mediation analysis. Path coefficients were estimated using the Bootstrap method, and the moderating effect was tested with the *PROCESS* macro in *SPSS*.

**Results:**

The study found that DSM significantly enhances the PRS of individuals through two pathways: “PIC → CSC → ERS” and “SIF → PCSM → PRC.” The PRS further positively predicts PS. In addition, DSDT plays a significant positive moderating role between DSM use and PRS.

**Conclusion:**

DSM plays a positive role in promoting the PRS of rehabilitation patients. It not only provides informational and social support but also activates cognitive and emotional mechanisms, ultimately enhancing PS. The study confirms the feasibility of DSM as an intervention tool in digital rehabilitation and highlights the importance of individual behavioral characteristics.

## Background

Social media has become an important channel for accessing health information and psychological support (Sun et al., 2023; Naslund et al., 2020). For sports injury rehabilitation patients, beyond physical treatment, the PRS is directly related to rehabilitation quality and return to activity (Kamphoff et al., 2013). Although prior studies indicate that online social support can alleviate anxiety, enhance emotional regulation, and foster connectedness (Lu and Hsu, 2013; Yang et al., 2014), there is still a lack of systematic explanations of why and through which psychological pathways social media exerts its effects. Much of the existing evidence stays at the outcome level and rarely integrates the “information processing” and “social behavior” routes together with individual differences within a single framework (Ma and Sayama, 2014; Naslund et al., 2016; Whiting and Williams, 2013).

To address this gap, this study constructs a pathway model that integrates chain mediation and moderation, testing how social media enhances the PRS through two chained mechanisms—PIC → CSC → ERS, and SIF → PCSM → PRC. We further examine how these improvements strengthen PS, while also testing the moderating role of DSDT. This design aims to provide testable theoretical grounding and practical guidance for digital interventions in sports rehabilitation.

## Digital social media and psychological recovery state

In the process of sports injury rehabilitation, individuals face not only the recovery of physical function but also the reconstruction of their psychological state (Wiese-Bjornstal et al., 1998). PRS refers to the overall level of emotional balance, energy restoration, and cognitive clarity after experiencing physical or emotional trauma. It is an important psychological indicator for evaluating the quality of rehabilitation (Clement and Arvinen-Barrow, 2013).

With the widespread use of social media, DSM has become one of the primary channels through which sports injury rehabilitation patients acquire rehabilitation information, express emotions, and seek social support. Relevant studies have pointed out that the higher the frequency of DSM use, the more emotional support and psychological comfort users receive, which may directly enhance their PRS (Fiedler et al., 2024). Through mechanisms such as instant information access, autonomous expression, and emotional feedback, DSM not only helps alleviate feelings of loneliness and anxiety among rehabilitation patients but may also strengthen their confidence in the rehabilitation process and foster positive emotional responses, thereby directly promoting psychological recovery.

Therefore, as a continuous psychological support environment, DSM may have a significant positive impact on the PRS of sports injury rehabilitation patients. Although previous studies have preliminarily confirmed its potential value, the specific mechanisms through which it functions in the context of sports injury rehabilitation still require further systematic validation and theoretical exploration.

## Chain mediation of perceived information credibility, coping strategy change, and emotional recovery speed

As an important platform for information acquisition, DSM not only provides abundant health-related resources but also influences individuals' PIC during psychological processing. Previous studies have indicated that factors such as the authority, consistency, and accuracy of content on social media significantly affect users' PIC, which in turn influences their cognitive and emotional responses (Flanagin and Metzger, 2007; Appelman and Sundar, 2016).

When individuals perceive the health information they receive as credible, they are more likely to adopt effective CSC, such as actively seeking social support, regulating emotions proactively, or employing problem-focused strategies, thereby improving their psychological adaptability (Carver et al., 1989; Folkman and Moskowitz, 2004). In the context of sports rehabilitation, such adjustments in coping strategies not only help individuals view their injuries more rationally but also buffer the stress and anxiety brought about during the rehabilitation process.

In summary, DSM may enhance users' PIC regarding health content, which in turn triggers positive CSC, accelerates the process of emotional recovery, and ultimately contributes to a better PRS. This chain of effects reflects a typical psychological mechanism of information processing → coping transformation → emotional regulation, providing a theoretical foundation for understanding the indirect psychological support that DSM offers to rehabilitation patients.

## Chain mediation of social interaction frequency, perceived companionship on social media, and perceived recovery control

In the process of sports injury rehabilitation, individuals require not only physiological treatment interventions but also face emotional distress, feelings of loneliness, and uncertainty about the recovery process. As a highly interactive media platform, DSM demonstrates unique value in providing social connection and psychological support to rehabilitation patients. Among its components, the frequency of social interaction on the platform has become one of the key initiating variables for psychological recovery. It reflects how frequently individuals engage in social behaviors such as commenting, sharing, and private messaging (Oh and Lee, 2012; Fiedler et al., 2024).

A higher frequency of social interaction helps enhance individuals' PCSM—that is, the sense of empathy, emotional support, and social belonging received from others through the platform. This process not only reduces the subjective sense of isolation among rehabilitation patients but also fosters their motivation to engage in platform interaction and emotional expression, forming a perceived “state of connectedness” (Clark et al., 2018; Voggenreiter et al., 2023).

Based on the enhancement of PCSM, rehabilitation patients are more likely to develop confidence and a sense of control over their recovery process. This is manifested in their subjective perception of control and understanding regarding the pace of treatment, physical condition, and rehabilitation plans, namely, PRC (Wallston et al., 1978). As a key mediating variable in improving the PRS, PRC not only reduces feelings of helplessness during rehabilitation but also strengthens individuals' motivation to persist in target behaviors and maintain emotional stability. Therefore, PRC, as a form of subjective cognitive resource, enhances individuals' mastery over the recovery process and directly contributes to the overall improvement of PRS by stabilizing emotions and sustaining recovery motivation.

In summary, DSM promotes the overall PRS of rehabilitation patients by stimulating SIF, enhancing PCSM, and improving PRC. This pathway may constitute a chain mediation process from social behavior to social support and ultimately to recovery-related cognition.

## The moderating effect of digital self-disclosure tendency

Although previous studies have confirmed the positive role of DSM in psychological recovery through information dissemination, social support, and access to rehabilitation resources, individual differences in platform behavior may significantly influence the extent to which users benefit from these functions. Among these differences, DSDT serves as a key moderating variable in this process (Ma et al., 2016; Lapidot-Lefler and Barak, 2015).

DSDT refers to an individual's willingness and frequency to actively express personal information, emotional states, and rehabilitation progress on social media (Taddicken, 2014). In the context of sports injury rehabilitation, individuals with a higher DSDT are more likely to share their emotional experiences and recovery journeys through social platforms, thereby gaining more emotional support and informational feedback. This interactive process not only strengthens their social connection but also facilitates emotional release and self-regulation, ultimately enhancing their PRS (Zhang et al., 2023).

In contrast, for individuals with a lower DSDT, even when exposed to abundant content on DSM, their limited level of engagement and the lack of effective channels for expression and feedback may weaken the platform's role in emotional regulation and cognitive recovery. Therefore, DSDT may moderate the pathway through which DSM use influences PRS, by either strengthening or diminishing the effectiveness of social media interventions.

In summary, this study designates DSDT as a moderating variable, aiming to examine its marginal regulatory role in the process by which DSM influences PRS, thereby further revealing the mechanism through which individual differences affect the rehabilitation pathway.

## Psychological recovery state and platform stickiness

In the context of digital health, an individual's continued use of a platform depends not only on the functions it provides but also on the psychological benefits gained during its use. PRS reflects the overall level of emotional balance, energy restoration, and cognitive clarity following physical injury. As the recovery state improves, individuals are more likely to develop a positive psychological affiliation with the platform, thereby enhancing PS (Forbes et al., 2023; Fiedler et al., 2024).

PS refers to users' willingness and behavioral dependence to continuously use a given platform, which is reflected in dimensions such as usage frequency, time spent, functional attachment, and emotional connection (O'Brien and Toms, 2008). In this study, PRS significantly predicts PS, indicating that when users perceive positive psychological recovery feedback through DSM, they are more likely to strengthen their usage loyalty and dependence on the platform (Fiedler et al., 2024).

Furthermore, the enhancement of PS not only extends users' interaction time with the platform but also encourages them to continue seeking rehabilitation-related information and support through DSM in the future, thereby reinforcing the platform's value in psychological recovery. This process constitutes a user experience feedback loop: DSM promotes psychological recovery → improved psychological recovery increases PS → increased PS in turn drives continued use of social media, forming a positive cycle of “platform attachment—psychological benefit—sustained use.”

Therefore, understanding the relationship between PRS and PS contributes to expanding the explanatory framework of how psychological experience influences technology usage stickiness, and provides theoretical support for designing digital platforms with stronger rehabilitation-oriented functions.

## Research questions, model, and hypotheses

**Figure 1:**
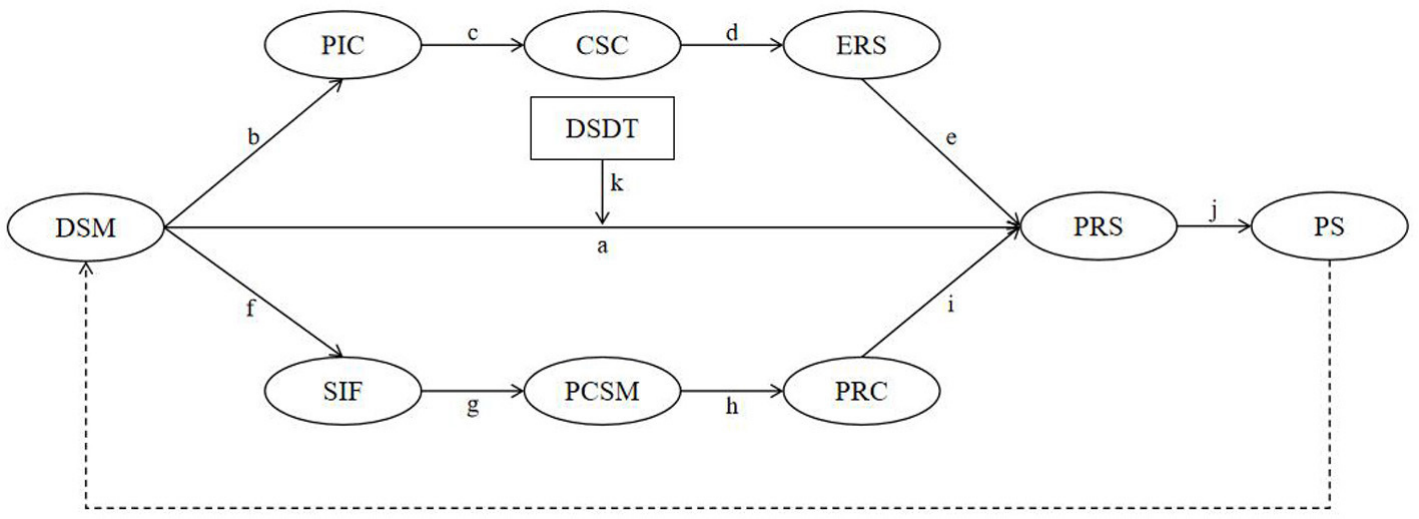
Conceptual model of chain mediation and moderation. DSM, digital social media; PIC, perceived information credibility; CSC, coping strategy change; ERS, emotional recovery speed; SIF, social interaction frequency; PCSM, perceived companionship on social media; PRC, perceived recovery control; PRS, psychological recovery state; PS, platform stickiness; DSDT, digital self-disclosure tendency; effect “*aj*” = DSM → PRS → PS; effect “*bcdej*” = DSM → PIC → CSC → ERS → PRS → PS; effect “*fghij*” = DSM → SIF → PCSM → PRC → PRS → PS.

Building on the above theoretical analysis and empirical findings, this study asks: (1) whether DSM enhances the PRS of sports injury rehabilitation patients via the chained mechanism PIC → CSC → ERS; (2) whether DSM enhances the PRS via the chained mechanism SIF → PCSM → PRC; (3) whether the PRS positively predicts PS (usage frequency, time spent, functional attachment, and emotional connection); and (4) whether DSDT moderates the pathway through which DSM influences the PRS. To address these questions, we construct the research model shown in Figure 1 and propose research hypotheses H1–H4.

**H1:** DSM use has an indirect effect on PS through PRS, forming the pathway “DSM → PRS → PS” (path “*aj*”).

**H2:** DSM influences PRS through PIC (path “*b*”), CSC (path “*c*”), and ERS (path “*d*”), which then affects PS (path “*j*”), forming a full chain mediation pathway: “DSM → PIC → CSC → ERS → PRS → PS” (path “*bcdej*”).

**H3:** DSM enhances PRS through SIF (path “*f* ”), PCSM (path “*g*”), and PRC (path “*h*”), which in turn influences PS (path “*j*”), forming another chain mediation pathway: “DSM → SIF → PCSM → PRC → PRS → PS” (path “*fghij*”).

**H4:** DSDT significantly moderates the relationship between DSM and PRS, such that the positive effect of DSM on PRS is stronger when the level of DSDT is higher (path “*k”* as the moderation path).

## Conceptual definitions

To ensure conceptual clarity, measurement consistency, and theoretical precision, the key constructs examined in this study are defined as follows:

***Digital Social Media (DSM):*** Digital Social Media refers to internet-based and mobile platforms that enable users to browse information, engage in social interaction, and generate user-created content. In the present study, DSM is conceptualized as a multifunctional digital environment through which sports injury rehabilitation patients obtain rehabilitation-related information, engage in social interaction, and express emotional experiences. Its core functions include information acquisition, social connection, and emotional support.

***Perceived Information Credibility (PIC):*** Perceived Information Credibility refers to individuals' subjective evaluation of the trustworthiness of information in terms of its authority, accuracy, and consistency. In the rehabilitation context, PIC reflects patients' judgments regarding whether health- and rehabilitation-related information encountered on digital social media is reliable, and it serves as a critical antecedent of information processing and subsequent coping behaviors.

***Coping Strategy Change (CSC):*** Coping Strategy Change refers to the process by which individuals adjust or transform their coping approaches when facing stressors or challenging situations. In this study, CSC captures rehabilitation patients' shifts from maladaptive or avoidant coping toward more adaptive strategies, such as problem-focused coping, emotion regulation, and active seeking of social support, influenced by their engagement with DSM.

***Emotional Recovery Speed (ERS):*** Emotional Recovery Speed refers to the rate and efficiency with which individuals return to emotional equilibrium following negative emotional experiences or stressful events. In the context of sports injury rehabilitation, ERS reflects patients' capacity to recover from emotions such as pain-related distress, frustration, or anxiety, and constitutes a core component of psychological recovery.

***Social Interaction Frequency (SIF):*** Social Interaction Frequency refers to the extent to which individuals engage in interactive behaviors on digital social media platforms, including commenting, liking, sharing, private messaging, and content posting. SIF represents users' level of social participation within the platform and serves as the initiating variable of the social behavior pathway in the proposed model.

***Perceived Companionship on Social Media (PCSM):*** Perceived Companionship on Social Media refers to individuals' subjective sense of being understood, cared for, and emotionally accompanied through their interactions on digital social media. This construct emphasizes perceived emotional presence and social support rather than the objective quantity of social interactions.

***Perceived Recovery Control (PRC):*** Perceived Recovery Control refers to individuals' beliefs regarding their ability to influence and manage their rehabilitation process, including control over physical condition, regulation of rehabilitation pace, and participation in recovery-related decision-making. In this study, PRC is conceptualized as a key cognitive resource linking social support to psychological recovery outcomes.

***Psychological Recovery State (PRS):*** Psychological Recovery State refers to the overall level of psychological functioning achieved after physical injury, encompassing emotional stability, energy restoration, and cognitive clarity. PRS serves as a central indicator of rehabilitation quality and is one of the core outcome variables in the present study.

***Platform Stickiness (PS):*** Platform Stickiness refers to users' tendency to continue using a digital platform and their degree of behavioral and emotional attachment to it. It is typically manifested through higher usage frequency, longer session duration, and stronger functional and emotional dependence. In this study, PS is conceptualized as a behavioral outcome resulting from psychological recovery benefits.

***Digital Self-Disclosure Tendency (DSDT):*** Digital Self-Disclosure Tendency refers to individuals' willingness and frequency of sharing personal information, emotional states, and lived experiences within digital social media environments. In the rehabilitation context, DSDT reflects patients' openness in expressing recovery-related experiences and emotions and represents a critical individual-difference factor moderating the effectiveness of DSM-based psychological support.

## Methods

**Figure 2:**
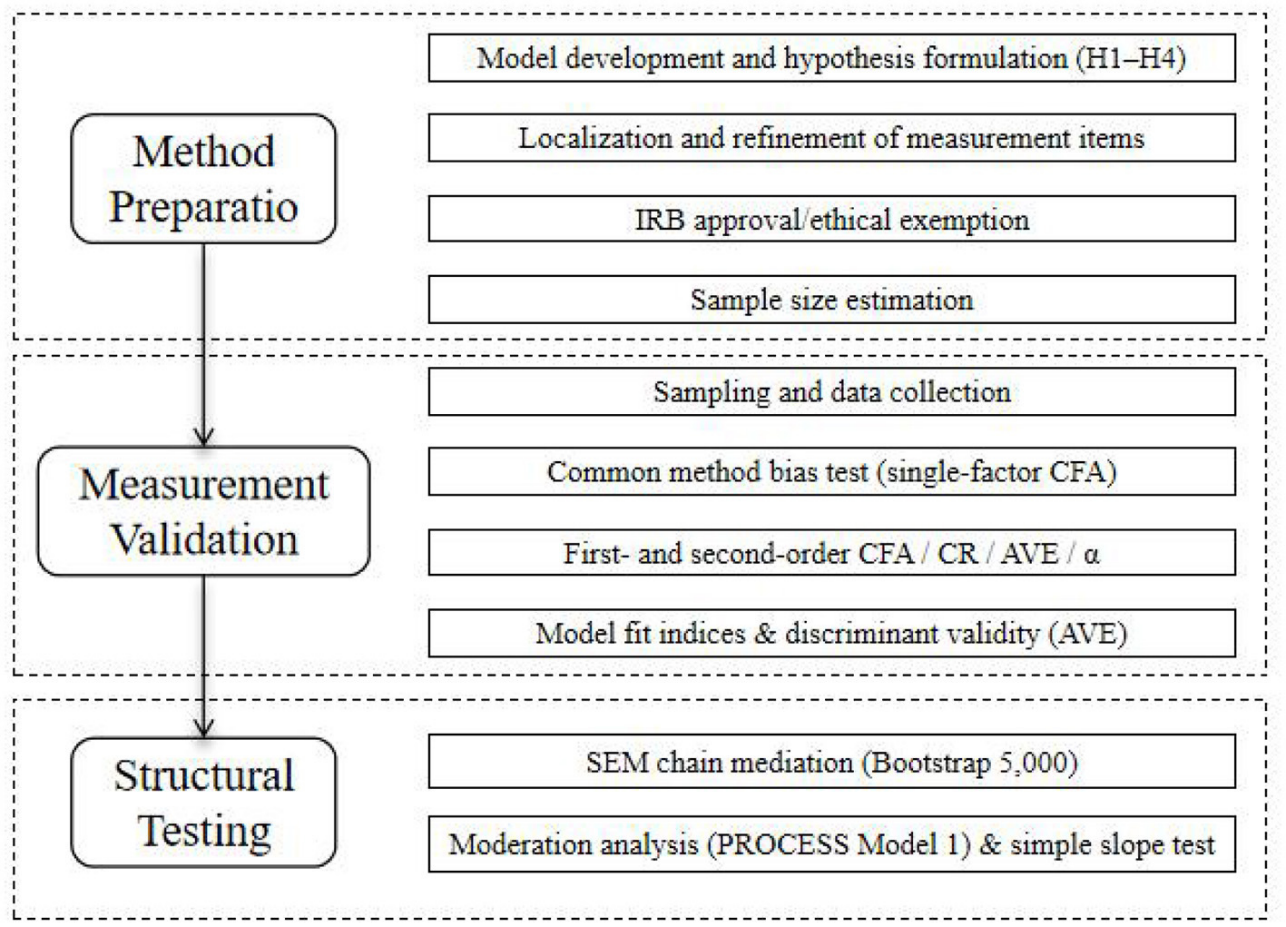
Research flow of the study. DSM, digital social media; PIC, perceived information credibility; CSC, coping strategy change; ERS, emotional recovery speed; SIF, social interaction frequency; PCSM, perceived companionship on social media; PRC, perceived recovery control; PRS, psychological recovery state; PS, platform stickiness; DSDT, digital self-disclosure tendency.

To provide a clear overview of the research procedure, the overall study process was organized into three sequential stages: method preparation, measurement validation, and structural testing. Each stage contained specific steps, ranging from hypothesis formulation and instrument refinement to confirmatory factor analyses, reliability and validity testing, and the final structural equation modeling. The detailed research flow is illustrated in Figure 2.

### Sample size estimation

The sample size was estimated using the formula proposed by (Kish 1965), denoted as $n=\frac{Z^{2}\cdot p\cdot \left(1-p\right)}{d^{2}}$, which has been widely applied in psychological research. The parameters used were: *Z* = 1.96 (the critical value of the normal distribution for a 95% *CI*), *p* = 0.5 (the expected proportion), and *d* = 0.04 (the allowable margin of error). Based on this calculation, the estimated sample size was approximately 600.

### Participants

A random sampling method was used to collect data from sports injury rehabilitation patients in 36 cities across 15 provincial-level regions in China. The survey was distributed via an online platform, aiming to maximize regional diversity and improve the representativeness of the sample. Data collection ended on April 16, 2025, yielding a total of 613 responses. To ensure data quality, the survey was completed anonymously, and each participant received a reward of 5 RMB. After screening for invalid responses—including duplicate submissions and clearly unreliable answers-−17 questionnaires were excluded, resulting in 596 valid responses, with a valid response rate of 97.23%.

The final sample consisted of 596 sports injury rehabilitation patients aged 18 years and older. Among them, 307 were male (51.5%), with an average age of 28.19 years (*SD* = 3.96), and the average age of female participants was 28.52 years (*SD* = 2.93). The primary DSM platforms used by participants included Douyin, WeChat Channels, Weibo, Xiaohongshu, Zhihu, and Bilibili, which are widely used for accessing health information, emotional expression, and sharing rehabilitation experiences.

Regarding sports background, event type was primarily individual sports (358, 60.1%) with team sports secondary (238, 39.9%); the competitive level was predominantly lower level (384, 64.4%), with higher level accounting for 212 (35.6%). For DSM use and interaction, 401 (67.3%) reported frequent use/interaction and 195 (32.7%) occasional use/interaction. Injury sites were most commonly the knee (214, 35.9%) and ankle (173, 29.0%), followed by the lumbar–back region (120, 20.2%) and shoulder (89, 14.9%). With respect to sport-psychology support, 318 (53.4%) received psychoeducation and 278 (46.6%) did not; 287 (48.2%) engaged in emotion-regulation training and 309 (51.8%) did not; and 254 (42.6%) reported frequent peer/coach support or professional psychological counseling, whereas 342 (57.4%) reported occasional support.

### Measurement instruments

A total of 10 measurement scales were used in this study. All scales were adapted based on established theoretical frameworks and validated instruments from prior research, with localization and semantic adjustments made to fit the research context. In accordance with the requirements of second-order construct modeling, the reliability and validity of each dimension were optimized and integrated. All scales were constructed as second-order scales, as detailed below:

Digital social media: composed of three second-order dimensions—browsing, interaction, and usage—with a total of 10 first-order items (Lee and Ma, 2012; Whiting and Williams, 2013); Perceived information credibility: comprised of three second-order dimensions—authority, accuracy, and consistency—with a total of 10 first-order items (Flanagin and Metzger, 2007; Appelman and Sundar, 2016); Coping strategy change: included three second-order dimensions—problem-focused coping, emotional regulation, and seeking social support—with a total of 9 first-order items (Carver et al., 1989; Folkman and Moskowitz, 2004); Emotional recovery speed: composed of three second-order dimensions—emotion recognition, recovery response, and time perception—with a total of 10 first-order items (Tugade and Fredrickson, 2004; Ong et al., 2006); Social interaction frequency: consisted of four second-order dimensions—active online communication, online interaction, friend circle sharing, and platform engagement frequency—with a total of 11 first-order items (Wellman and Gulia, 2018; Valkenburg and Peter, 2009); Perceived companionship on social media: composed of five second-order dimensions—online companionship, sense of being understood, perceived empathic support, emotional connection, and social belonging—with a total of 16 first-order items (Whiting and Williams, 2013; Naslund et al., 2016); Perceived recovery control: comprised of three second-order dimensions—control over physical condition, management of rehabilitation pace, and belief in autonomous decision-making—with a total of 16 first-order items (Wallston et al., 1978; Mullan et al., 2013); Psychological recovery state: consisted of three second-order dimensions—emotional stability, energy restoration, and cognitive clarity—with a total of 10 first-order items (Kellmann and Kallus, 2001; Lu et al., 2016); Platform stickiness: included four second-order dimensions—intention to reuse, time spent, functional dependence, and emotional connection—with a total of 12 first-order items (O'Brien and Toms, 2008; Limayem et al., 2007); Digital self-disclosure tendency: composed of four second-order dimensions—willingness to share information, willingness to express emotions, sense of control, and social motivation—with a total of 13 first-order items (Taddicken, 2014).

The Cronbach's α coefficients for the ten measurement scales used in this study ranged from 0.920 to 0.959, indicating excellent internal consistency reliability. In addition, all scales passed confirmatory factor analysis (*CFA*), with model fit indices meeting statistical standards, demonstrating good structural validity and measurement stability across constructs. These results confirm the appropriateness of the scales for the present research context. Detailed reliability and validity statistics are presented in Table 1.

**Table 1:** Fit indices of the latent variable measurement models.

**Variables**	***χ^2^***	***df***	***χ^2^/df***	***GFI***	***AGFI***	***CFI***	***NFI***	***RMSEA***
DSM	35.074	32	1.096	0.988	0.980	0.999	0.990	0.013
PIC	44.689	32	1.397	0.985	0.975	0.986	0.987	0.026
CSC	35.947	24	1.498	0.987	0.975	0.996	0.988	0.029
ERS	56.626	32	1.770	0.982	0.968	0.993	0.984	0.036
SIF	41.311	38	1.087	0.988	0.979	0.999	0.990	0.012
PCSM	94.244	94	1.003	0.981	0.972	0.999	0.985	0.002
PRC	86.558	101	0.857	0.983	0.977	0.999	0.985	0.001
PRS	30.477	32	0.952	0.990	0.983	0.999	0.994	0.002
PS	45.402	48	0.946	0.988	0.980	0.999	0.993	0.001
DSDT	80.410	59	1.363	0.980	0.969	0.995	0.983	0.025

DSM, digital social media; PIC, perceived information credibility; CSC, coping strategy change; ERS, emotional recovery speed; SIF, social interaction frequency; PCSM, perceived companionship on social media; PRC, perceived recovery control; PRS, psychological recovery state; PS, platform stickiness; DSDT, digital self-disclosure tendency.

### Data analysis

A range of statistical methods was employed to ensure the reliability and validity of the measurement model and to systematically test the significance of both chain mediation and moderation effects (with the significance level set at *p* < 0.05).

First, to address potential common method bias (*CMB*), a single-factor *CFA* was conducted using *AMOS 26.0*. All measurement items were loaded onto a single latent factor to construct a one-factor model, which was used to assess the extent of *CMB* in the dataset.

Second, based on the first-order model, *CFA* was performed on the ten latent variables. Items with factor loadings below 0.45 were removed, and second-order models were constructed accordingly. The resulting second-order models demonstrated satisfactory fit indices, further supporting the structural validity of the measurement model.

Next, *IBM SPSS 26.0* was used to conduct correlation and descriptive statistical analyses to examine the preliminary relationships and distribution characteristics among the variables. To further test the chain mediation effects, structural equation modeling (*SEM*) was conducted using *AMOS 26.0*. The chain mediation paths were pre-coded in the backend, and the Bootstrap method (with 5,000 resampling iterations) was applied to estimate bias-corrected confidence intervals for each path coefficient, thereby testing the three hypothesized chain mediation pathways.

For the moderation analysis, the *PROCESS* macro (*Model 1*) was used in *SPSS* to perform regression analysis, testing the moderating role of DSDT in the relationship between DSM use and PRS. To enhance visualization of the interaction effects, simple slope plots were generated using the output of *PROCESS* and graphically presented with Microsoft PowerPoint (Version 2019) to assist in interpreting the moderation effect.

## Results

### Common method bias and model fit evaluation

To assess whether common method bias existed in this study, a single-factor *CFA* was conducted using *AMOS*, following the approach suggested by Podsakoff et al. (2003). All measurement items were loaded onto a single latent factor to construct a one-factor model. The results indicated poor model fit: χ^2^ = 12,750.348, *df* = 434, χ^2^*/df* = 29.379, *GFI* = 0.377, *AGFI* = 0.288, *CFI* = 0.361, *NFI* = 0.354, and *RMSEA* = 0.218. These values did not meet the model fit criteria recommended by Hu and Bentler (1999): χ^2^*/df* ≤ 3, *GFI, AGFI, CFI*, and *NFI* ≥ 0.90, and *RMSEA* ≤ 0.06. This suggests that a single factor could not account for the variance among all measured variables.

Subsequently, a full measurement model including all latent variables was tested. The results demonstrated a good model fit: χ^2^ = 479.178, *df* = 424, χ^2^*/df* = 1.130, *GFI* = 0.951, *AGFI* = 0.942, *CFI* = 0.997, *NFI* = 0.976, and *RMSEA* = 0.015. Therefore, the results indicate that common method bias is not a serious concern in this study.

In addition to the overall measurement model, separate confirmatory factor analyses were conducted for each construct to ensure their individual reliability and validity. Additional results are provided in Supplementary Table 1.

### Confirmatory factor analysis

Based on the first-order model, *CFA* was conducted using *AMOS* for all observed variables. Following Brown's (2015) recommendations, items with standardized factor loadings below 0.45 were removed, and the remaining variables were grouped to form a second-order model. The results showed that the standardized factor loadings of all measurement variables in the second-order model ranged from 0.842 to 0.956 and were all statistically significant (*p* < 0.001), indicating strong correlations between the observed variables and their corresponding latent constructs.

In addition, the composite reliability (*CR*) values for all latent constructs ranged from 0.920 to 0.959, and the average variance extracted (*AVE*) values ranged from 0.793 to 0.879, indicating good internal consistency and convergent validity of the measurement instruments. These results meet the criteria proposed by Fornell and Larcker (1981), which state that standardized factor loadings should exceed 0.50, *CR* should be greater than 0.60, and *AVE* should not be lower than 0.40 when *CR* is high.

Therefore, it can be concluded that the constructs in this study demonstrate satisfactory reliability and convergent validity, and exhibit strong measurement stability. The detailed CFA results for each latent variable are presented in Table 2.

**Table 2:** Confirmatory factor analysis results.

**Latent variable**	**Observed variable**	***Unstd*.**	***S.E*.**	***Z***	***p***	***Std*.**	***SMC***	***CR***	***AVE***	***Cronbach's* α**
DSM	DSM1	1.000				0.898	0.807	0.929	0.813	0.920
	DSM2	1.004	0.031	32.237	^***^	0.902	0.814			
	DSM3	1.307	0.040	32.359	^***^	0.904	0.817			
PIC	PIC1	1.000				0.929	0.864	0.926	0.807	0.926
	PIC2	0.954	0.030	31.974	^***^	0.876	0.768			
	PIC3	0.944	0.029	32.852	^***^	0.889	0.790			
CSC	CSC1	1.000				0.881	0.776	0.920	0.793	0.92
	CSC2	1.044	0.035	29.971	^***^	0.899	0.808			
	CSC3	0.995	0.034	29.650	^***^	0.892	0.795			
ERS	ERS1	1.000				0.921	0.848	0.926	0.806	0.925
	ERS2	0.973	0.031	31.464	^***^	0.876	0.768			
	ERS3	1.013	0.031	32.740	^***^	0.896	0.802			
SIF	SIF1	1.000				0.894	0.800	0.942	0.802	0.941
	SIF2	1.025	0.030	34.119	^***^	0.911	0.830			
	SIF3	1.042	0.030	34.730	^***^	0.918	0.843			
	SIF4	1.014	0.034	29.866	^***^	0.858	0.736			
PCSM	PCSM1	1.000				0.871	0.758	0.954	0.807	0.954
	PCSM2	1.031	0.033	30.841	^***^	0.889	0.790			
	PCSM3	1.036	0.032	32.096	^***^	0.905	0.820			
	PCSM4	1.029	0.031	33.452	^***^	0.923	0.851			
	PCSM5	1.047	0.033	31.869	^***^	0.903	0.815			
PRC	PRC1	1.000				0.941	0.885	0.948	0.858	0.948
	PRC2	0.993	0.024	41.132	^***^	0.928	0.860			
	PRC3	0.974	0.025	39.053	^***^	0.911	0.829			
PRS	PRS1	1.000				0.931	0.866	0.956	0.879	0.956
	PRS2	1.001	0.022	45.281	^***^	0.956	0.914			
	PRS3	0.993	0.024	41.177	^***^	0.926	0.858			
PS	PS1	1.000				0.929	0.864	0.959	0.854	0.959
	PS2	0.997	0.025	40.114	^***^	0.919	0.845			
	PS3	1.009	0.026	39.562	^***^	0.915	0.838			
	PS4	1.022	0.024	42.045	^***^	0.933	0.870			
DSDT	DSDT1	1.000				0.916	0.840	0.939	0.794	0.937
	DSDT2	1.001	0.030	33.391	^***^	0.883	0.779			
	DSDT3	1.013	0.034	29.932	^***^	0.842	0.709			
	DSDT4	1.015	0.027	37.116	^***^	0.921	0.848			

DSM, digital social media; PIC, perceived information credibility; CSC, coping strategy change; ERS, emotional recovery speed; SIF, social interaction frequency; PCSM, perceived companionship on social media; PRC, perceived recovery control; PRS, psychological recovery state; PS, platform stickiness; DSDT, digital self-disclosure tendency.

^***^p < 0.001.

### Correlation and descriptive statistical analysis

As shown in the descriptive statistics in Table 3, the mean scores (*M*) of the ten latent variables ranged from 3.960 to 4.030, indicating that all latent constructs, from DSM to sports performance, were evaluated positively. The absolute values of skewness ranged from 0.016 to 0.081, and those of kurtosis ranged from 0.532 to 0.952. These values did not exceed the commonly accepted thresholds for normality—absolute skewness less than two and absolute kurtosis less than seven—suggesting that the data approximated a normal distribution (George, 2011).

**Table 3:** Correlation and descriptive statistics.

**Variables**	**1**	**2**	**3**	**4**	**5**	**6**	**7**	**8**	**9**	**10**
1. DSM	***0.902***									
2. PIC	0.369^***^	***0.898***								
3. CSC	0.134^***^	0.259^***^	***0.891***							
4. ERS	0.044^*^	0.082^**^	0.341^***^	***0.898***						
5. SIF	0.362^***^	0.114^***^	0.045^*^	0.042^*^	***0.896***					
6. PCSM	0.123^***^	−0.042^*^	0.006^*^	0.001^*^	0.302^***^	***0.898***				
7. PRC	0.078^*^	0.087^**^	0.032^*^	−0.027^*^	0.075^*^	0.279^***^	***0.926***			
8. PRS	0.339^***^	0.166^***^	0.100^**^	0.195^***^	0.121^***^	0.141^***^	0.495^***^	***0.938***		
9. PS	0.331^***^	0.179^***^	0.095^**^	0.194^***^	0.118^***^	0.141^***^	0.504^***^	0.957^***^	***0.924***	
10. DSDT	0.100^**^	0.071^*^	−0.003^*^	0.008^*^	0.098^**^	0.004^*^	0.007^*^	0.004^*^	0.015^*^	***0.891***
*M*	3.980	4.000	3.960	3.990	4.000	3.980	4.010	4.030	4.030	3.990
*SD*	1.525	1.153	1.137	1.148	1.177	1.117	1.105	0.809	0.810	1.471
*Skewness*	−0.016	0.034	0.045	0.024	−0.059	−0.062	0.026	0.056	0.033	−0.081
*Kurtosis*	−0.932	−0.640	−0.716	−0.779	−0.585	−0.667	−0.823	0.532	0.563	−0.952

^***^p < 0.001, ^**^p < 0.01, ^*^p < 0.05; The bold italic values represent the square roots of the AVE on the diagonal.

DSM, digital social media; PIC, perceived information credibility; CSC, coping strategy change; ERS, emotional recovery speed; SIF, social interaction frequency; PCSM, perceived companionship on social media; PRC, perceived recovery control; PRS, psychological recovery state; PS, platform stickiness; DSDT, digital self-disclosure tendency.

Additionally, discriminant validity among the ten variables was assessed using the square roots of the *AVE*. The results showed that the square root of each construct's *AVE* was greater than its correlations with other constructs, indicating satisfactory discriminant validity (Hair et al., 2010).

### Chain mediation effect analysis

**Figure 3:**
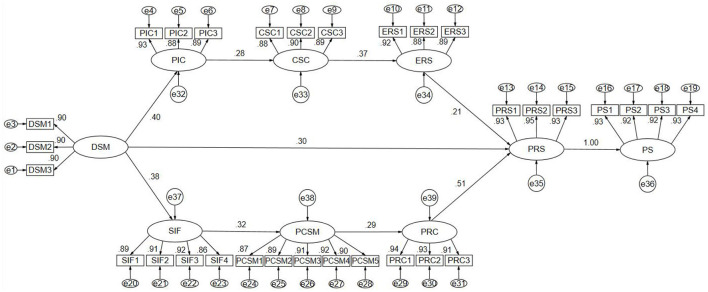
Chain mediation path diagram. DSM, digital social media; PIC, perceived information credibility; CSC, coping strategy change; ERS, emotional recovery speed; SIF, social interaction frequency; PCSM, perceived companionship on social media; PRC, perceived recovery control; PRS, psychological recovery state; PS, platform stickiness; DSDT, digital self-disclosure tendency; effect “*aj*” = DSM → PRS → PS; effect “*bcdej*” = DSM → PIC → CSC → ERS → PRS → PS; effect “*fghij*” = DSM → SIF → PCSM → PRC → PRS → PS.

Figure 3 presents the standardized path coefficients of the chain mediation model. To enhance the completeness of the results, the unstandardized path coefficients output from *AMOS* are also reported as follows: The path from DSM to PRS (path “*a*”) was γ = 0.162, *p* < 0.001. The path from DSM to PIC (path “*b*”) was γ = 0.308, *p* < 0.001. The path from PIC to CSC (path “*c*”) was γ = 0.267, *p* < 0.001. The path from CSC to ERS (path “*d*”) was γ =0.379, *p* < 0.001. The path from ERS to PRS (path “*e*”) was γ = 0.146, *p* < 0.001. The path from DSM to SIF (path “*f* ”) was γ = 0.291, *p* < 0.001. The path from SIF to PCSM (path “*g*”) was γ = 0.301, *p* < 0.001. The path from PCSM to PRC (path “*h*”) was γ = 0.297, *p* < 0.001. The path from PRC to PRS (path “*i*”) was γ = 0.367, *p* < 0.001. Finally, the path from PRS to PS (path “*j*”) was γ = 0.993, *p* < 0.001.

The chain mediation effects were analyzed and tested using structural equation modeling to enhance the accuracy of computation. First, the Bootstrap method was applied to estimate the standard errors of the chain mediation effects and to assess their significance. The results (Table 4) showed that the total effect of the model was 0.175, with a standard error (*SE*) of 0.022. The absolute value of the *Z*-score (*Z* = *Mean/SE*) was 7.955, exceeding the critical value of 1.96. The *95% CI* [0.132, 0.219] excluded zero, *p* < 0.01, indicating that the total effect was statistically significant. Specifically:

**Table 4:** Chain mediation effects.

**Paths and hypotheses**	**Chained mediation**	**Effect**	Product of coefficients		Bias-corrected ***95% CI***		***p***
			***SE***	***Z***	**Lower**	**Upper**	
H1 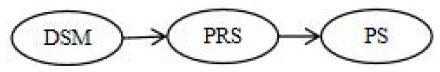	Effect “aj”	0.161	0.022	7.318	0.118	0.206	0.0004
H2 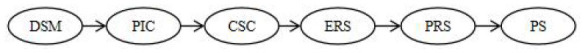	Effect “bcdej”	0.005	0.001	5.000	0.003	0.007	0.0002
H3 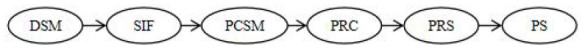	Effect “fghij”	0.010	0.002	4.500	0.015	0.015	0.0003
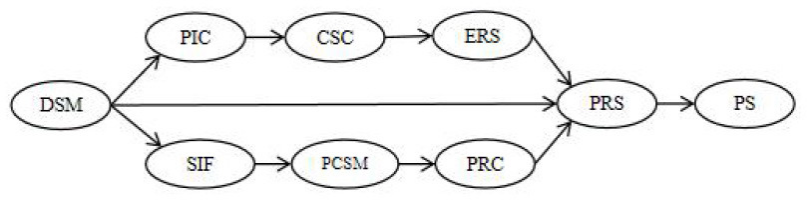	Total model	0.175	0.022	7.955	0.132	0.219	0.0004

DSM, digital social media; PIC, perceived information credibility; CSC, coping strategy change; ERS, emotional recovery speed; SIF, social interaction frequency; PCSM, perceived companionship on social media; PRC, perceived recovery control; PRS, psychological recovery state; PS, platform stickiness; DSDT, digital self-disclosure tendency; effect “aj” = DSM → PRS → PS; effect “bcdej” = DSM → PIC → CSC → ERS → PRS → PS; effect “fghij” = DSM → SIF → PCSM → PRC → PRS → PS.

**H1** (effect “*aj*”), the estimated chain mediation effect was 0.161, *SE* = 0.022, *Z* = 7.318, with a bias-corrected *95% CI* [0.118, 0.206], *p* = 0.0004. Since the confidence interval did not include zero and the Z-score was significant, the chain mediation effect of H1 was supported. **H2** (effect “*bcdej*”), the estimated chain mediation effect was 0.005, *SE* = 0.001, *Z* = 5.000, with a bias-corrected *95% CI* [0.003, 0.007], *p* = 0.0002, supporting the significance of the H2 chain mediation pathway. **H3** (effect “*fghij*”), the estimated chain mediation effect was 0.010, *SE* = 0.002, *Z* = 4.500, with a bias-corrected *95% CI* [0.006, 0.015], *p* = 0.0003, also confirming the significance of the H3 mediation path.

### Moderation effect analysis

To examine the moderating role of DSDT in the relationship between DSM and PRS, a regression model including the interaction term was constructed. Both the moderating variable and its interaction with the independent variable were entered into the analysis. To further illustrate the nature of the moderation effect, a simple slope plot was generated and reported.

According to the results in Table 5, the overall model demonstrated good fit (*F*(3, 592) = 61.586, *p* < 0.001), explaining 23.8% of the total variance in PRS (*R*^2^ = 0.238). DSM use had a significant positive predictive effect on PRS (β = 0.187, *t* = 9.768, *p* < 0.001, *95% CI* [0.150, 0.225]). The moderating variable, DSDT, did not have a significant direct effect on PRS (β = −0.005, *t* = −0.24, *p* > 0.05, *95% CI* [−0.044, 0.034]).

**Table 5:** Moderation effect.

**Variable type**	**Variable**	Dependent variable: PRS			
		β	*SE*	*t*	*95% CI*
Independent variable	DSM	0.187^***^	0.019	9.768	[0.150, 0.225]
Moderating variable	DSDT	−0.005	0.02	−0.240	[−0.044, 0.034]
Interaction term (H4)	DSM × DSDT (effect “k”)	0.133^***^	0.014	9.741	[0.106, 0.159]
	*R*	0.487			
	*R^2^*	0.238			
		F(3, 592) = 61.586^***^			

^***^p < 0.001; PRS, psychological recovery state; DSM, digital social media; DSDT, digital self-disclosure tendency.

More importantly, the interaction term (effect “*k*”) between DSM and DSDT significantly predicted PRS (β = 0.133, *t* = 9.741, *p* < 0.001, *95% CI* [0.106, 0.159]), indicating that DSDT exerted a significant positive moderating effect on this pathway, thereby supporting Hypothesis H4.

**Figure 4:**
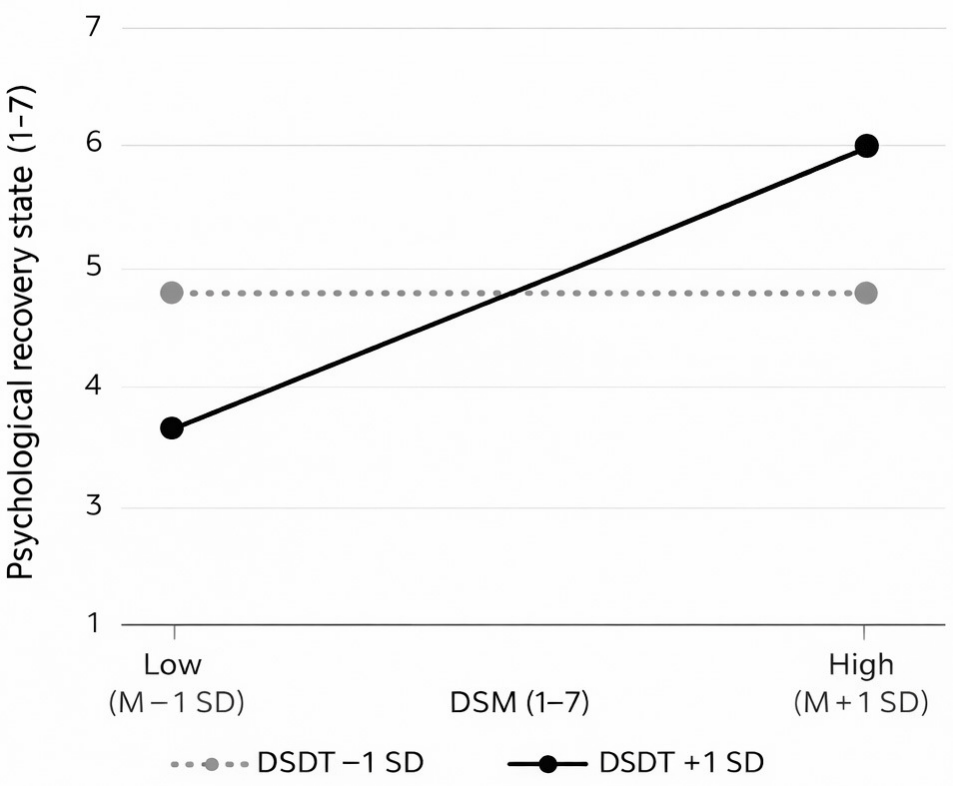
Simple slope plot. DSM, digital social media; DSDT, digital self-disclosure tendency. Low = M −− 1 SD; High = M + 1 SD, where M is the mean of DSM scores (centered on the scale 1–7).

Figure 4 illustrates the simple slope analysis for the moderation effect. The results show that when the moderating variable DSDT is at a low level (−1 *SD*), the predictive effect of DSM on PRS is not significant (with a slope close to zero). In contrast, when DSDT is at a high level (+1 *SD*), the predictive effect becomes significantly stronger (with a noticeably steeper slope), further confirming Hypothesis H4.

## Discussion

This study constructed and tested a moderated chain mediation model to explain how DSM contributes to the PRS and PS among sports injury rehabilitation patients. The results confirmed two significant mediation pathways and a feedback loop mechanism. In addition, DSDT was found to moderate the relationship between DSM use and psychological recovery. These findings provide a comprehensive understanding of both the psychological benefits and behavioral consequences of DSM use in the recovery process.

### Psychological mechanism of the information processing chain

The first mediation pathway reveals the sequential mediating roles of PIC, CSC, and ERS in the process through which DSM influences PRS. This mechanism reflects a “cognitive appraisal” perspective, aligning with Lazarus and Folkman (1984), which posits that individuals' appraisal of information determines the type of coping strategies they adopt, ultimately affecting their emotional and psychological outcomes. Previous studies have suggested that when individuals perceive health information as authoritative, accurate, and consistent, they develop clearer expectations regarding the rehabilitation process, making them more likely to adopt active, problem-focused coping strategies (Appelman and Sundar, 2016; Carver et al., 1989).

Subsequently, changes in coping strategies further influence individuals' emotional regulation and recovery pace. High-quality coping strategies can reduce anxiety and feelings of helplessness, enabling individuals to exhibit greater ERS when facing pain or rehabilitation stress (Tugade and Fredrickson, 2004). As a core dimension of PRS, emotional recovery not only affects one's current rehabilitation status but also determines persistence and self-efficacy in future recovery behaviors (Bandura and Locke, 2003). Therefore, this study validates a four-stage pathway—PIC → CSC → ERS → *psychological recovery*—highlighting that the functional role of social media in enhancing psychological recovery lies not only in information dissemination but also in shaping the psychological adaptation process.

### Rehabilitation mechanism of the social behavior chain

The second mediation pathway highlights the role of social interaction and emotional support in the rehabilitation process. A high frequency of social interaction indicates that individuals engage more frequently in communication, expression, and feedback-seeking on digital platforms. This interactive process fosters a strong sense of “connectedness” and “presence” (Voggenreiter et al., 2023), thereby enhancing PCSM. During rehabilitation, perceived companionship serves as a proxy for emotional support, effectively reducing feelings of loneliness, frustration, and uncertainty throughout the recovery journey (Ma and Sayama, 2014).

Building on this, the perceived social support and empathic understanding gained from interactions enhance the individual's sense of PRC—that is, their belief in their ability to manage the pace of rehabilitation, control their physical condition, and participate in recovery-related decision-making (Wallston et al., 1978). This subjective sense of control not only boosts confidence in recovery but also significantly improves PRS (Thoits, 2011). In other words, social interaction is not only a fundamental feature of social media but also a bridge to emotional relief and behavioral autonomy. From the perspective of the social behavior pathway, this study empirically supports the sequential mechanism of *social participation → emotional connection* → PRC, emphasizing the critical role of the social value of digital platforms in fostering psychological resilience during rehabilitation.

### Feedback mechanism from psychological recovery state to platform stickiness

Beyond the chain mediation pathways, the study also revealed that PRS strongly predicts PS. This finding underscores the importance of the “psychological benefit–continued use” feedback model in technology usage (O'Brien and Toms, 2008). When individuals gain positive experiences such as emotional relief, cognitive clarity, and energy restoration from platform use, they are more likely to develop greater trust and reliance on the platform, which in turn increases their willingness to continue using it, extend session durations, or revisit more frequently. This positive feedback loop supports the user experience theory's proposition that *perceived benefits* directly influence *usage stickiness*, providing psychological evidence for designing social platforms with integrated rehabilitation support functions (Fiedler et al., 2024).

### Moderating role of digital self-disclosure tendency

Finally, the moderation analysis revealed that DSDT significantly and positively moderated the relationship between DSM use and PRS. Specifically, individuals with a high tendency toward self-disclosure are more likely to proactively express their recovery status, share rehabilitation experiences, and display emotional states on social platforms. This openness makes it easier for them to receive external feedback, social support, and psychological responses, thereby accelerating their recovery process. This finding aligns with Ong et al. (2006) theory that self-disclosure enhances social gains and further extends the moderating function of social behavior in rehabilitation contexts.

In contrast, individuals with a low tendency toward self-disclosure may have limited engagement on the platform, as they are less willing or accustomed to expressing their emotions and health conditions. As a result, they are less likely to receive effective interactive feedback, which weakens the impact of DSM on their psychological recovery, consistent with prior research showing that lower self-disclosure and engagement are associated with reduced motivational activation and diminished benefits from digital health interventions (Deci and Ryan, 2000; Taddicken, 2014; Naslund et al., 2016). This suggests that whether individual traits can be transformed into recovery resources within digital platforms depends more on users' motivation to participate and their expressive habits than on the platform's functional features. These findings highlight the need for digital platforms aiming to facilitate rehabilitation to balance users' willingness to disclose with appropriate privacy management strategies, thereby strengthening the positive cycle of “expression–feedback–recovery” (Luo and Hancock, 2020).

## Theoretical implications

This study theoretically extends the applicability of DSM within the domain of psychological health interventions by systematically exploring its impact on the psychological recovery of rehabilitation patients. By constructing a model that integrates *dual chain mediation and moderation mechanisms*, the study demonstrates that DSM facilitates psychological recovery not through a single channel, but through two distinct pathways: the “PIC → coping strategy → emotional recovery” route and the “social interaction → perceived companionship → PRC” route. This model synthesizes perspectives from health communication theory, coping theory, and social connection theory, addressing previous gaps in the understanding of psychological rehabilitation mechanisms. Additionally, by introducing DSDT as a moderating variable, the study highlights the influence of individual behavioral styles on intervention effectiveness, uncovering the interactive dynamics between personal traits and platform mechanisms. These findings offer new theoretical support for the advancement of digital psychology and personalized health interventions.

## Practical implications

This study offers important insights for sports rehabilitation practices and digital health interventions. First, the findings confirm that DSM can serve as an auxiliary psychological support tool in the rehabilitation process. Platform developers may utilize the identified pathway mechanisms to design features that enhance recovery functions—such as personalized recommendations, emotion diaries, and rehabilitation information prompts. Second, the study highlights the moderating role of DSDT, suggesting that intervention strategies should be tailored to individual differences. For users with high willingness to express, platforms can incorporate open interaction and experience-sharing functions; for those with low expressive tendencies, features such as anonymous participation and non-verbal feedback can be adopted to reduce the psychological threshold and user pressure. Finally, the significant predictive effect of PRS on PS indicates that platforms capable of delivering meaningful psychological benefits can effectively improve user loyalty and engagement. Thus, rehabilitation institutions and product designers should prioritize users' emotional experiences and recovery feedback within the platform to promote a positive cycle of *recovery* → *stickiness* → *continued use*.

## Limitations

Although this study contributes to both theoretical development and empirical validation, several limitations should be acknowledged. First, the study employed a cross-sectional design based on a one-time questionnaire survey, which limits the ability to infer causal relationships or observe temporal dynamics among variables. Future research may adopt longitudinal tracking or experimental designs to strengthen causal inferences. Second, although the sample covered 15 provinces across China, data collection was primarily conducted through online questionnaires, which may have resulted in an overrepresentation of individuals who prefer using DSM, potentially affecting the representativeness and external validity of the sample. Third, all study variables were measured using self-reported scales. Although common method bias was statistically controlled, it remains difficult to completely eliminate the possibility of social desirability effects or response biases. Finally, although the moderating variable—DSDT—showed significant effects, its measurement dimensions were relatively broad. Future studies may refine the categorization of expression types (e.g., text-based vs. image-based disclosure) or explore additional individual-level moderators, such as social motivation or privacy anxiety, to further advance understanding of individual differences in digital recovery contexts.

## Future research

Based on the findings and limitations of this study, future research may be expanded in the following directions. First, it is recommended to adopt longitudinal or experimental designs to dynamically track changes in the psychological states of sports injury rehabilitation patients during their use of DSM, thereby enhancing the explanatory power for causal relationships. Second, future studies could incorporate physiological indicators (e.g., heart rate variability, sleep quality) or third-party behavioral tracking data (e.g., platform usage logs) to enrich measurement diversity and reduce self-report bias. Third, while this study focused on DSDT as a moderating variable, future research could explore additional individual difference variables such as social motivation, privacy concerns, and self-presentation strategies to better understand how they influence the effect pathways of digital platforms on psychological recovery. Moreover, comparisons between different platform types (e.g., information-oriented vs. emotion-oriented) could clarify the complex interaction mechanisms among platform attributes, user characteristics, and recovery pathways. Finally, cross-cultural validation using diverse cultural samples is recommended to improve the generalizability and theoretical applicability of the proposed model.

## Conclusion

This study aimed to investigate how DSM facilitates the PRS of sports injury rehabilitation patients through multiple psychological mechanisms and, in turn, enhances PS. Based on data from 596 rehabilitation patients, the findings revealed that DSM not only directly improves PRS but also exerts indirect effects through two chain mediation pathways: PIC → CSC → ERS and SIF → PCSM → PRC. Moreover, PRS significantly and positively predicted PS, supporting the feedback mechanism of *psychological benefit → continued use*. Additionally, DSDT significantly moderated the above pathways, indicating that individual behavioral traits play a crucial role in the recovery process. These findings not only enrich the theoretical framework connecting DSM and psychological recovery mechanisms but also offer new pathways and tools for rehabilitation intervention practices. In conclusion, DSM can serve as a low-cost and highly accessible means of psychological support, playing a vital role in the context of sports rehabilitation. Future research should continue to explore its underlying mechanisms and expand its practical applications across diverse recovery settings.

## Data Availability

The datasets presented in this study can be found in online repositories. The names of the repository/repositories and accession number(s) can be found in the article/Supplementary material.
